# Chestnut!!

**Published:** 1887-01

**Authors:** 


### CHESTNUT!!

### A FROTHY MANIFESTO.

It is possible that but few physicians have perused the pages of THE HOMŒOPATHIC PHYSICIAN for August, since it may be that the circulation of that small, but by no means modest, magazine is not commensurate with its somewhat violent assumption of superiority. For the benefit of those who may never have heard of this sheet, we will give an exceedingly brief biographical sketch, for which we trust our contemporary will manifest a decent gratitude. It is not an onerous task to attempt, for there is but little to relate. Its origin is somewhat obscure. Evidence as to the necessity of its birth is entirely lacking. But these, perhaps, are minor matters of negation. The journal occupies its own particular place in the medical world. It is quite alone in its chosen field, for its plane is so narrow that it would be extremely difficult for any one else but its editor to effect a lodgement there. It is the organ of the "true healer," and its editors, also "true healers," grind away with a persistence worthy of a better cause. Its mission seems to be to publish monthly accounts of the great virtues and wisdom of "true healers," and bewail the fact that all wisdom will go with it to the grave, and to consign to perdition all who do not fall down and worship it. Should the picture just drawn appear the reverse of attractive, the reply is that the fault is in the original, and our justification for presenting it at all lies in the fact that this periodical, while attacking homœopathic enterprises and vilifying homœopathic workers, masquerades as THE HOMŒOPATHIC PHYSICIAN. In the August number, replying to a pleasant and courteous letter from Dr. Lilienthal, urging the journal to consider the error of its ways, its editor sputters forth the following abuse of the American Institute of Homœopathy: "In the early years of its existence the Institute was weak in numbers, but strong [the editor was a member] in men of ability and of purpose. In these latter years the Institute is strong in numbers, but lacking [the editor has deserted] in ability and purpose—in fact, it seems to have no purpose, unless a servile imitation of the allopath, and a ready adoption of each new fashion in medicine be evidence of a studied purpose to please the old school. About every principle which makes Homœopathy strong and victorious has been successfully denied and derided—all to please the allopath. The single dose is rejected; the minimum dose is ridiculed; every pathogenesis is placed upon a false basis, pathology. * * * In short, all the distinctive, all the useful, principles of Homœopathy are rejected to-day by nine-tenths of the members of the American Institute." Such frothy spleen as this is the legitimate out-growth of intolerance and prejudice, and merits contemptuous silence. For, speaking as mildly as possible, the extract quoted is nothing but a deliberate misrepresentation of facts. The printed transactions of the Institute contain the evidence which absolutely confutes every assertion so blatantly made. The Institute, as a deliberative body, has never rejected the single dose, nor has it ever ridiculed the minimum dose. The statement that all the distinctive principles of Homœopathy have been rejected carries its own refutation. The trouble with this editor is that he is a partisan myope, who will not put on spectacles and see either his own face in the mirror, or the world at large as others see them. All that is done inside the circle in which, by choice or circumstances, he finds himself placed, is rightly done. The pets of his selection can do no wrong. To exhibit their excellencies, to paint their superiorities, to cackle vicariously over their eggs, is one-half the business of his life. The other half is to cheapen, pick to pieces, ridicule, condemn, and, so far as he can, destroy the work of all outside the charmed line which circumscribes the area of his narrow sympathies. Within his field all questions are divine; sunflowers are suns, crab apples are pomegranates, and an onion is the fountain of tearful emotion. Outside of his field the land is desert and the people are barbarians, who not only do nothing well, but who are guilty of great presumption in attempting to do anything at all. These poisonous waters break on barren shores, and there dwell the graceless infidels who do not worship toward the holy hill where he has raised his tabernacle. Blinded by passion, he mistakes assertion for argument, coarse invective for energy, and deems common courtesy effeminacy. He calls on "Moses and the prophets." We advise him to peruse the epistles of Peter and Paul. Nobody will ever accuse them of weakness, yet though they never hesitated to declare "the whole counsel of God," and often thundered into unwilling ears the most disagreable truths, their epistles are as full of gentleness and of courtesy as they are of logic. It is as true as ever that it requires some talent and some generosity to find out generosity and talent in others, though nothing but self-conceit and malice are needed to discover or imagine faults. We would also meekly remind the sapient editor of THE HOMŒOPATHIC PHYSICIAN that the more readily we admit the possibility of our own cherished convictions being mixed with error, the more vital and helpful whatever is right in them will become, and no error is so conclusively fatal as the idea that the Almighty will not allow us to err, though He may allow other men to do so.—*North American Journal of Homœopathy, Oct.*, 1886, *page* 697.

